# Development and validation of prognostic model based on the analysis of autophagy-related genes in colon cancer

**DOI:** 10.18632/aging.203352

**Published:** 2021-07-27

**Authors:** Yongfeng Wang, Kaili Lin, Tianchun Xu, Liuli Wang, Liangyin Fu, Guangming Zhang, Jing Ai, Yajun Jiao, Rongrong Zhu, Xiaoyong Han, Hui Cai

**Affiliations:** 1 The First Clinical Medical College of Gansu University of Chinese Medicine, Lanzhou 730000, Gansu, China; 2 General Surgery Clinical Medical Center, Gansu Provincial Hospital, Lanzhou 730000, Gansu, China; 3 First Clinical Medical College, Lanzhou University, Lanzhou 730000, Gansu, China; 4 Graduate School, Ning Xia Medical University, Yinchuan 750004, Ning Xia, China; 5 Key Laboratory of Molecular Diagnostics and Precision Medicine for Surgical Oncology in Gansu Province, Gansu Provincial Hospital, Lanzhou 730000, Gansu, China; 6 Intelligent Medical Laboratory, Gansu Provincial Hospital, Lanzhou 730000, Gansu, China

**Keywords:** colon cancer, autophagy, gene signature, prognosis model, immune microenvironment

## Abstract

Background: Autophagy, a process of self-digestion, is closely related to multiple biological processes of colon cancer. This study aimed to construct and evaluate prognostic signature of autophagy-related genes (ARGs) to predict overall survival (OS) in colon cancer patients.

Materials and Methods: First, a total of 234 ARGs were downloaded via The Cancer Genome Atlas (TCGA) database. Based on the TCGA dataset, differentially expressed ARGs were identified in colon cancer. The univariate and multivariate Cox regression analysis was performed to screen prognostic ARGs to construct the prognostic model. The feasibility of the prognostic model was evaluated using receiver operating characteristic curves and Kaplan-Meier curves. A prognostic model integrating the gene signature with clinical parameters was established with a nomogram.

Results: We developed an autophagy risk signature based on the 6 ARGs (*ULK3, ATG101, MAP1LC3C, TSC1, DAPK1,* and *SERPINA1*). The risk score was positively correlated with poor outcome and could independently predict prognosis. Furthermore, the autophagy-related signature could effectively reflect the levels of immune cell type fractions and indicate an immunosuppressive microenvironment.

Conclusion: We innovatively identified and validated 6 autophagy-related gene signature that can independently predict prognosis and reflect overall immune response intensity in the colon cancer microenvironment.

## INTRODUCTION

Colon cancer, the common type of tumor in the digestive system, ranks as the third most common malignant tumor globally [1]. Colon cancer is related to miscellaneous factors, including diet, environment, genetic and epigenetic changes [2]. The occurrence of most colon cancers is related to chromosomal and microsatellite instability mechanisms [3]. The comprehensive therapy has made great progress in colon cancer; however, the long-term survival rate remains very low in advanced colon cancer patients [4]. Consequently, it is of necessity to screen out sensitive and specific molecular biomarkers to contribute to predicting the prognosis of this deadly disease and providing individualized efficient therapy guidance [5].

Depending on the environment, autophagy acts as a dual part in cancer [6], possibly reducing the viability of tumor cells or as a cytoprotective mechanism [7]. In some situations, autophagy can produce nutrients to provide energy for cells by constitutively eliminating defective proteins and organelles [8]. Conversely, autophagy can trigger cell death under certain conditions, such as over-regulation of autophagy or long-term exposure to autophagy [8]. Autophagy has played an important intermediary role in terms of resistance to radiation, chemotherapy, and targeted agents [9, 10].

The high-throughput platform has provided more facilitated genome exploration on different cancers including colon cancer for clinicians and bioinformatics. There is an increasing interest in studies on basis of the prognostic model containing lncRNA, miRNA and mRNA in recent years. For example, Lin et al. identified a new risk scoring model including 12 autophagy-related genes (ARGs) having great prognostic prediction value in breast cancer [11]. Du et al. reported a new risk scoring model including 5-ARGs, indicating great prognostic value for patients with breast cancer [12]. Zhou et al. reported a 10-lncRNA risk model in breast cancer, which showed significantly different survival outcomes in the different risk-groups [13], and a survival-associated module and RNA binding proteins were identified in invasive breast carcinoma, which contained lncRNA, miRNA, and mRNA simultaneously [14]. Nevertheless, little attention has been paid to the autophagy-related prognostic model in colon cancer. Therefore, to improve prognostic evaluation in colon cancer patients, this study first screened and identified OS-related ARGs based on the TCGA dataset, and further established an autophagy-related signature to develop novel therapeutic strategies in colon cancer.

## MATERIALS AND METHODS

### Data acquisition

A total of 232 ARGs were obtained via The Human Autophagy Database, including comprehensive information of human genes related to autophagy (http://autophagy.lu/clustering/index.html). The Cancer Genome Atlas (TCGA) data is public repository (https://cancergenome.nih.gov/). RNA sequence transcriptome data and clinical information for 473 colon cancer and 41 non-tumor counterparts were acquired via TCGA.

### Distinct expressed ARGs analysis and enrichment analysis

To identify the differentially expressed ARGs, we applied Wilcoxon test by “limma” R package. The thresholds for the differentially expressed ARGs were set to |log_2_ fold change (FC)|> 1 along with *P* < 0.05 for false discovery rate (FDR). Then, we performed Gene Ontology (GO) and Kyoto Gene and Genomic Encyclopedia (KEGG) pathway enrichment analyses to evaluate relevant biological function and pathways. The significance threshold of the output categories was set to *p* and *q* < 0.05. The histogram, bubble, and circle plot present annotation analysis results by applying the R package “enrichplot”, “GOplot”, and “ggplot2”.

### Construction of prognosis prediction model

We first carried out univariate Cox regression analyses to screen out ARGs having prognostic value in colon cancer. Subsequently, multivariate Cox regression was carried out in order to establish ARG signature using R “survival” package. We obtained the risk score of each patient with colon cancer according to the expression of predictive genes multiplied with the coefficients. The Kaplan-Meier (K-M) methods was implemented to evaluate the survival differences. The predictive value of ARG signature was estimated with the time-dependent receiver-operator characteristic (ROC) curve by R “survival ROC” package. Finally, principal component analysis (PCA) was implemented by the ‘prcomp’ method from the R ‘stats’ package, and t-distributed stochastic neighbor embedding (t-SNE) was implemented by the ‘Rtsne’ package.

### Assessment of the immune-/chemotherapeutic response

To evaluate the infiltration scores of immune cells along with immune- related functions between the different risk-groups, we implemented the single-sample gene set enrichment analysis (ssGSEA) and CIBERSORT algorithm (https://cibersort.stanford.edu/index.php). We analyzed the differences in the expression of Immune Checkpoint Inhibitors (ICIs) between the different risk-groups by Wilcoxon test using R ggpubr package. Correlation analysis implemented by Spearman methods was performed between the risk score and the expression of genes related to ICIs [15]. Moreover, based on the Genomics of Drug Sensitivity in Cancer (GDSC, https://www.cancerrxgene.org) [16], the difference of the half inhibitory centration (IC_50_) in the different risk-groups was analyzed by Wilcoxon test using pRRophetic and ggplot2 of R.

### Statistical analysis

All data processing was done in the R language (version 3.6). All statistical *P* values were two-tailed, with *p* < 0.05 as statistical significance.

### Ethical approval

This article does not contain any studies with human participants or animals performed by any of the authors.

## RESULTS

### Identification of distinct ARGs in non-tumor and colon cancer groups

**Figure 1:**
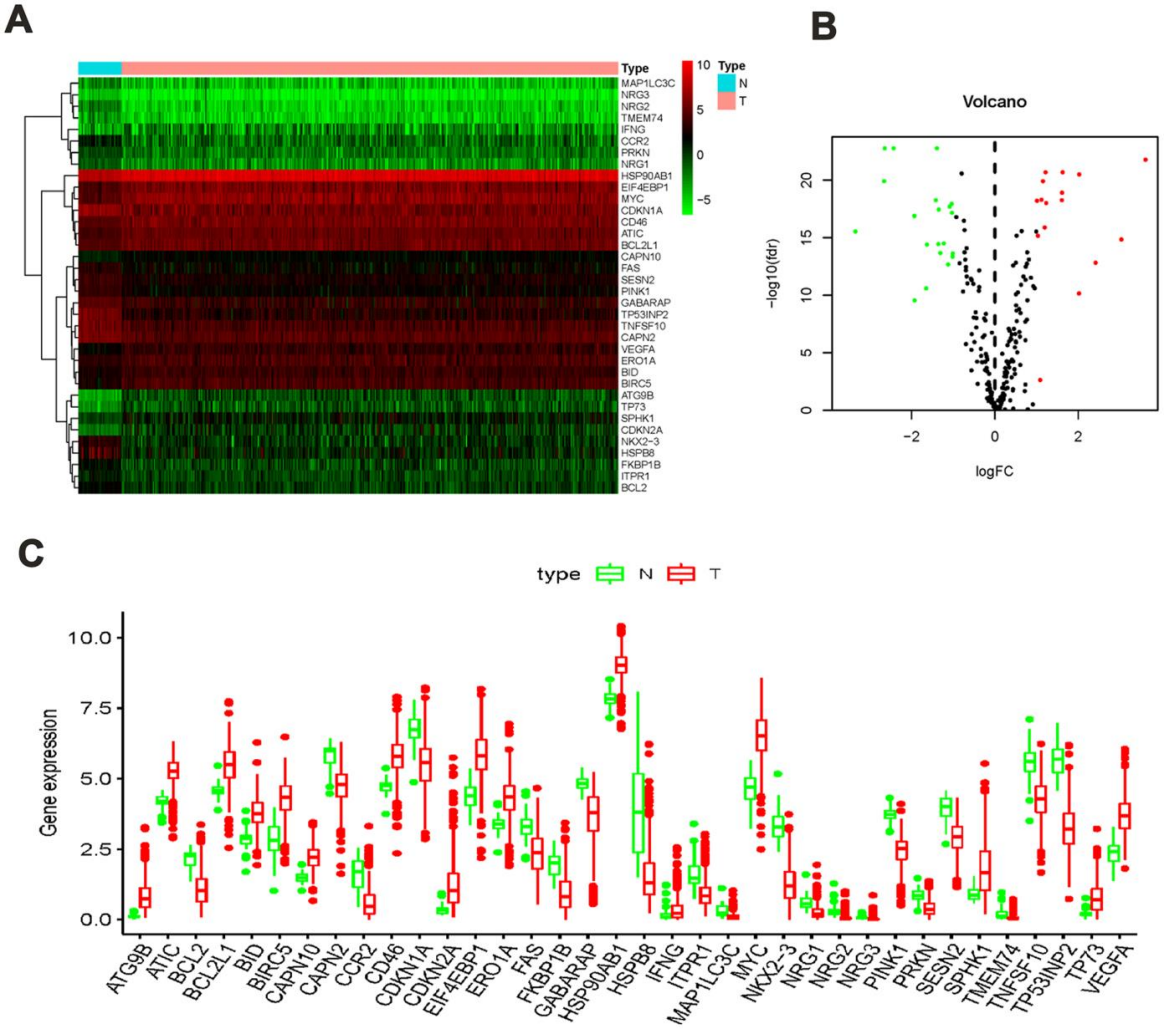
**ARGs differentially expressed in colon cancer and normal tissues.** (**A**) Heatmap showed thirty-six differentially expressed genes, with red dots indicating significantly up-regulated genes, green dots indicating significantly down-regulated genes, and black dots indicating no differences gene; (**B**) the volcanic map of differentially expressed genes; (**C**) the bar plot of genes in normal and tumor tissues. Red and green indicate tumor tissues and normal tissues.

A total of 41 non-tumor and 473 colon cancer tissues were extracted via TCGA data, which contains their RNA sequence transcriptome data and clinical information. We then extracted the expression data of 232 ARGs, among which, 36 significantly different ARGs were identified in non-tumor and colon cancer groups. The screening thresholds were set to |log_2_ (FC)|> 1 along with FDR <0.05. Compared with the normal group, 20 ARGs were found to be overexpression whereas 16 were found to be under-expression in the colon cancer group (Figure 1A, 1B). The detailed forecasting model establishment flowchart is shown in Supplementary Figure 1. In addition, the expression values of differentially expressed ARGs in non-tumor and colon cancer groups are shown in a bar plot (Figure 1C).

### Enrichment of the differentially expressed ARGs

**Figure 2:**
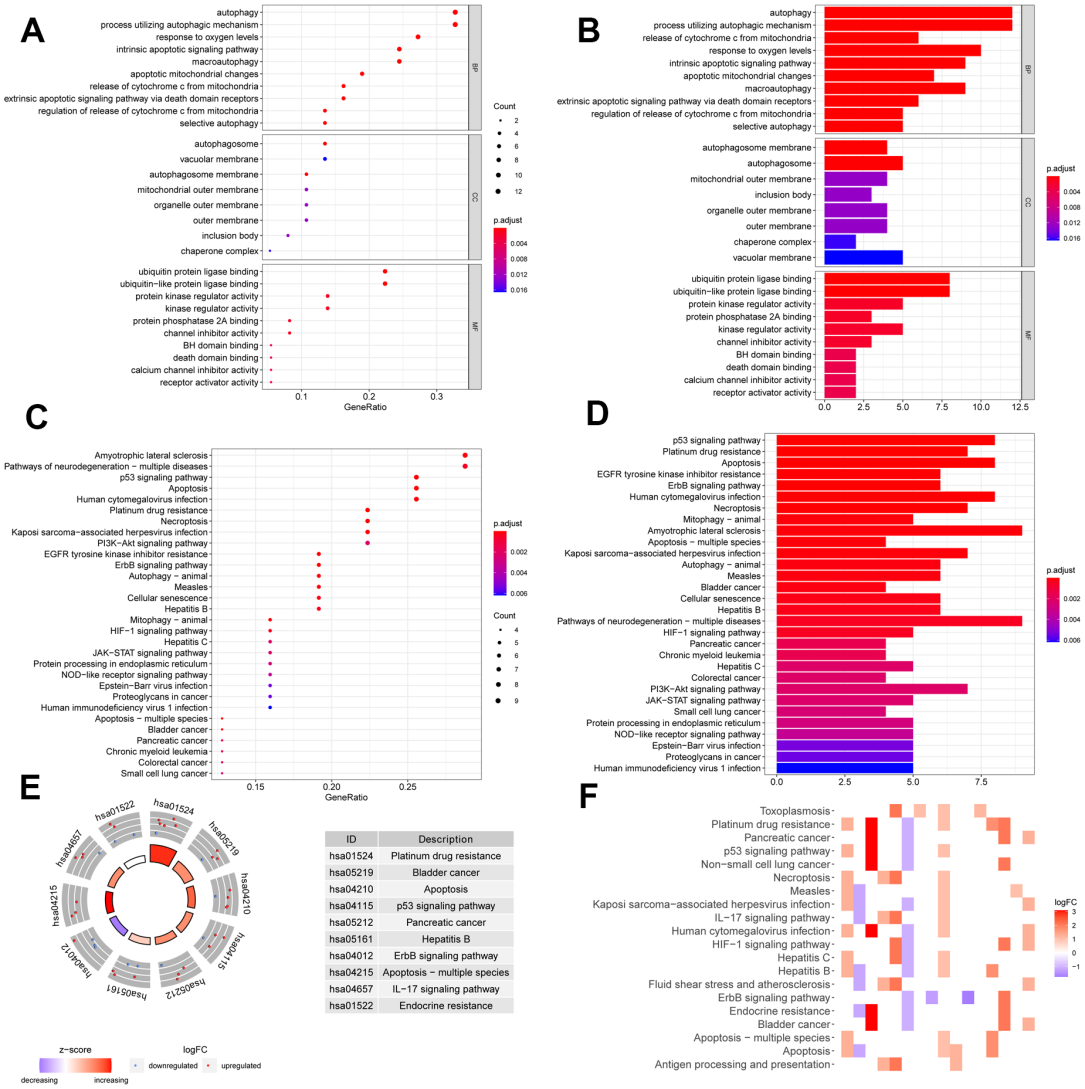
**Function annotation of differentially expressed ARGs.** Bubble chart (**A**) and histogram depiction (**B**) in the aspects of MF, BP, CC; bubble chart (**C**) and histogram depiction (**D**) of the top 30 enrichment pathways; (**E**) circle diagram of the top 10 significant enrichment pathways. The inner circle indicated Z-score. (**F**) The heatmaps of KEGG enrichment. The red color indicated the up- regulated genes and green represented down-regulated genes.

We performed GO and KEGG pathway enrichment of the differentially expressed ARGs to evaluate relevant biological function and pathways. In the GO enrichment analysis, the top 10 terms of molecular functions (MF), biological processes (BP), and cellular components (CC) are displayed in Figure 2A, 2B, including autophagy, processes utilizing autophagic mechanism, autophagosome, vacuolar membrane, ubiquitin protein ligase binding, protein kinase regulator activity, and other critical functions. In the pathway enrichment analysis, top 30 pathways are summarized in Figure 2C, 2D; they are mainly involved in p53 signaling pathway, apoptosis, mitophagy, necroptosis, *ErbB* signaling pathway, *EGFR* tyrosine kinase inhibitor resistance, and so on. KEGG heatmaps and circle plot also revealed the ARGs (Figure 2E, 2F).

### Identification of prognosis-related ARGs 22w

**Figure 3:**
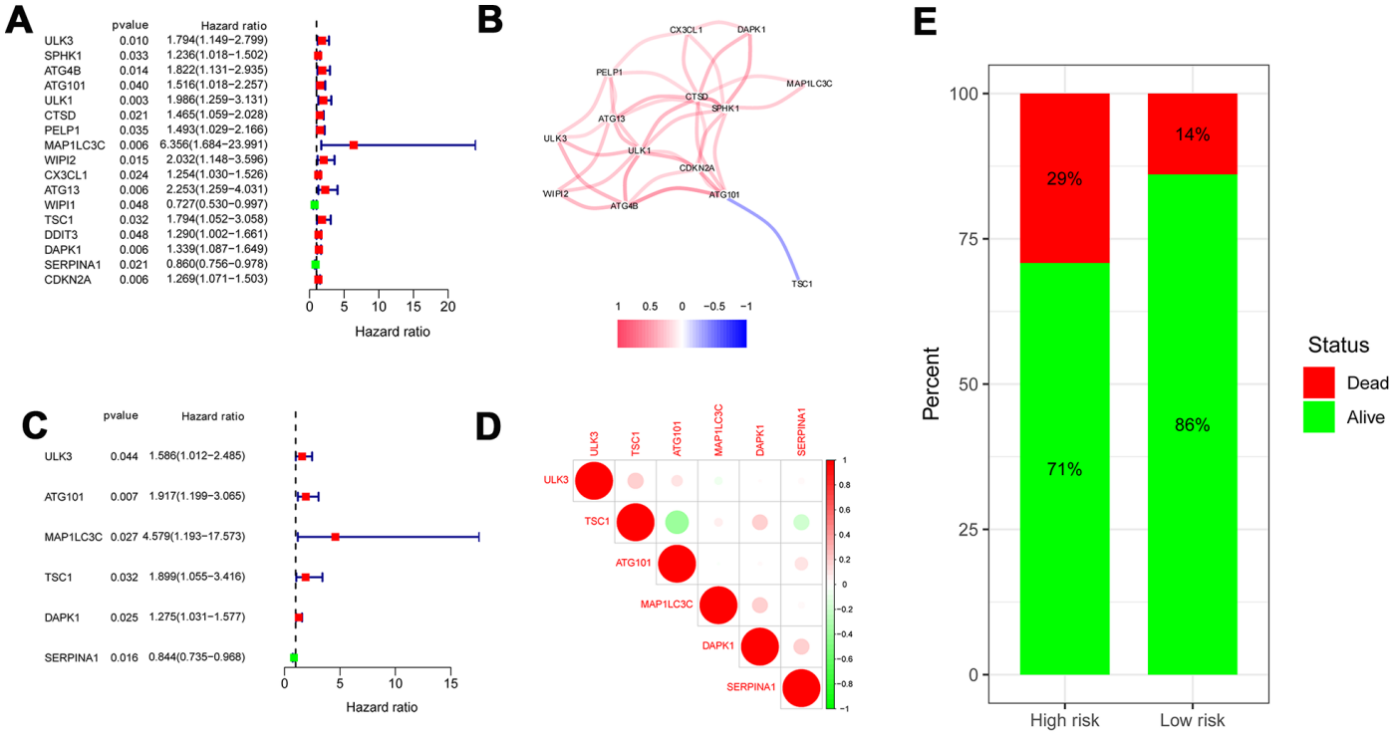
**Risk ratio forest plot showed the prognostic value of the OS-related ARGs.** (**A**) The forest plot of univariate Cox regression analysis. (**B**) The correlation network of OS-related ARGs. Correlation coefficients are represented by different colors. (red: positive correlations; blue: negative correlations). (**C**) The forest plot of multivariate Cox regression analysis. (**D**) Spearman correlation analysis of 6 hub genes in the TCGA databases. (**E**) Mortality rates of the low- and high- risk groups.

**Figure 4:**
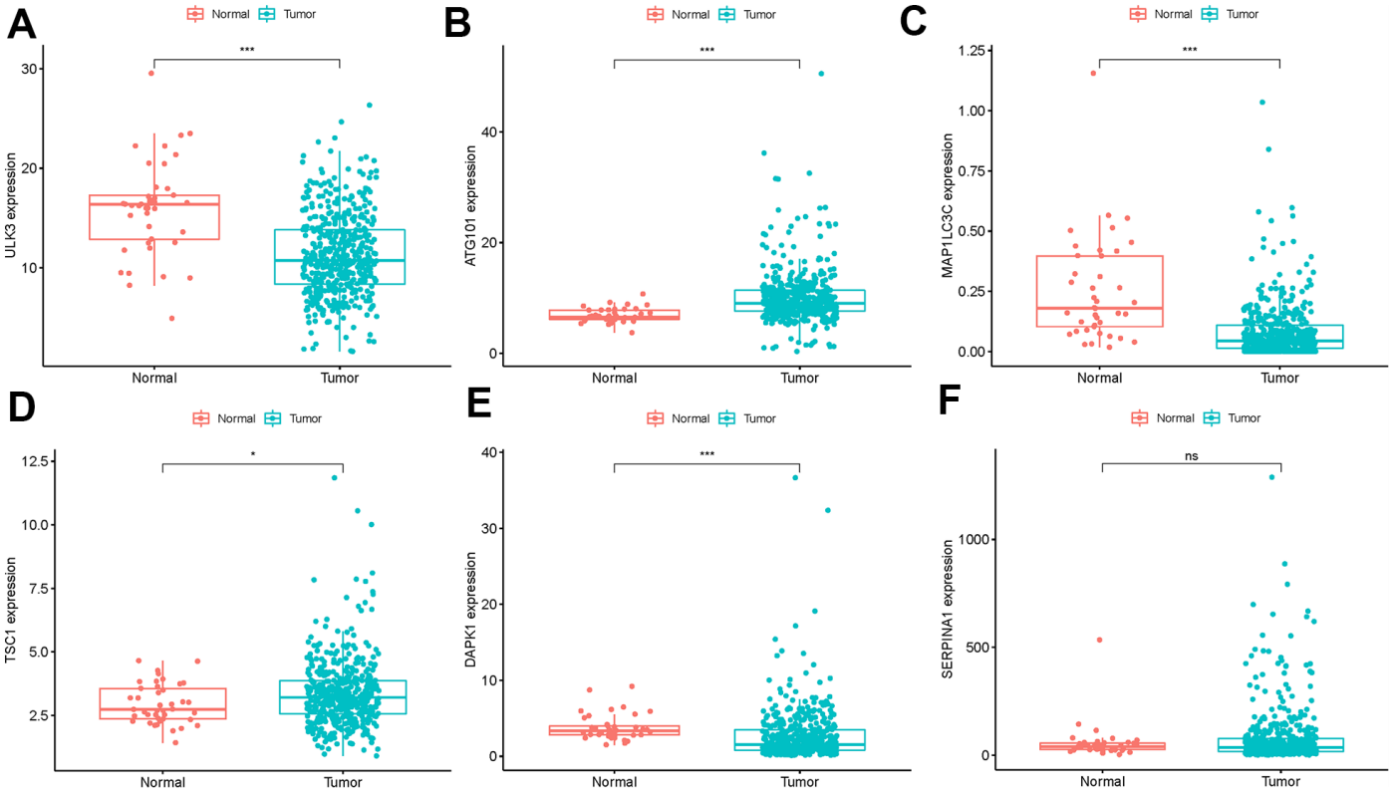
**Expression of the 6 ARGs in colon cancer (n=473) and normal samples (n=41) with unpaired t test.** (**A**) *ULK3*, (**B**) *ATG101*, (**C**) *MAP1LC3C*, (**D**) *TSC1*, (**E**) *DAPK1* (**F**) *SERPINA1* (*P<0.05, **P<0.01, ***P<0.001, ns:P>0.05).

We screened out ARGs significantly associated with OS under the cutoff value of *P* < 0.05 by univariate Cox regression analysis. The forest map shows that 14 ARGs were significantly related to survival rate (Figure 3A). A correlation network is summarized in Figure 3B on basis of the expression profiles of 14 OS-related ARGs. Furthermore, six genes including *ULK3*, *ATG101*, *MAP1LC3C*, *TSC1, DAPK1*, and *SERPINA1* were identified to establish autophagy-related prediction model through multivariate Cox regression analysis (Figure 3C and Table 1). Correlation analysis among 6 hub ARGs in TCGA using the Spearman method is summarized in Figure 3D. The differences of the 6 hub ARG expression in colon cancer and normal samples are displayed in Figure 4 by unpaired t test. In accordance with the median expression of 6 hub ARGs, the expression of *ATG101* (*P*=0.013), *DAPK1* (*P*=0.014), *SERPINA1* (*P*=0.032), *ULK3* (*P*=0.020) were significantly correlated with OS from colon cancer, whereas there was no correlation for MAP1LC3C (*P*=0.635) and TSC1 (*P*=0.150) as shown in Kaplan-Meier curves (Supplementary Figure 2).

**Table 1:** OS-related ARGs identified by multivariate Cox regression analysis.

**ARGs**	**coef**	**HR**	**Lower 95% CI**	**Upper 95% CI**	**P-value**
ULK3	0.46121	1.585992	1.012195	2.485065	0.044129
ATG101	0.650715	1.916912	1.19905	3.064553	0.006562
MAP1LC3C	1.521474	4.578972	1.193133	17.57305	0.026602
TSC1	0.641122	1.898611	1.055216	3.416098	0.032411
DAPK1	0.243022	1.275096	1.030898	1.577139	0.025057
SERPINA1	-0.17015	0.843537	0.734778	0.968394	0.015693

### Construction of prognosis prediction model

**Figure 5:**
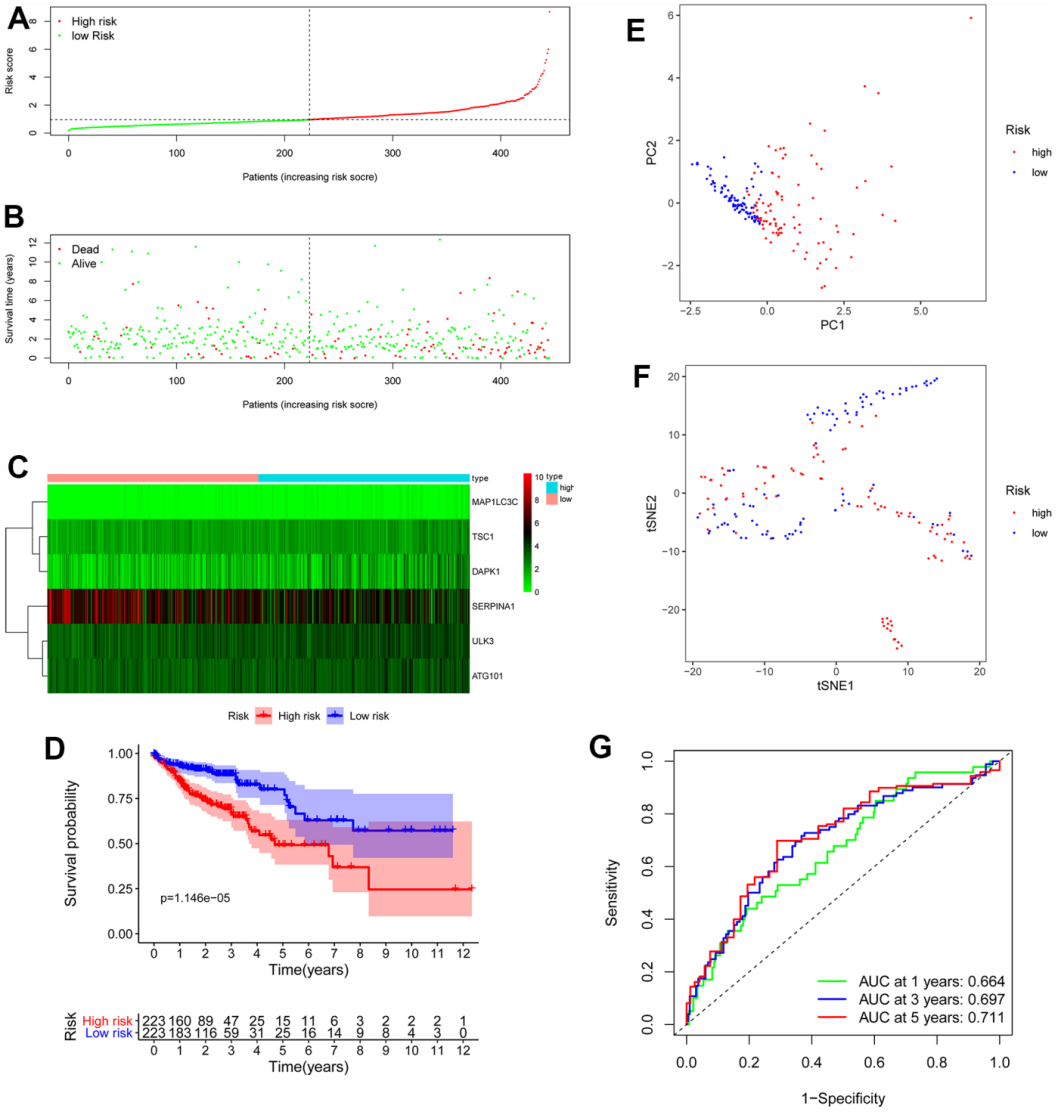
**OS-related prognostic model of colon cancer patients.** (**A**) The prognostic model distribution of colon cancer patients. (**B**) Survival status of patients in the TCGA dataset. (**C**) Heat map of the expression profile of the included ARGs. (**D**) The Kaplan-Meier survival curve showed that patients in the high-risk group have a significantly shorter overall survival. (**E**) Principal component analysis (PCA) plot. (**F**) T-distributed stochastic neighbor embedding (t-SNE) analysis. (**G**) Survival-dependent ROC curves validate the prognostic significance of ARGs-based prognostic indicators.

We obtained the risk score of each case with colon cancer; risk score = (0.46121 × expression of *ULK3*) + (0.650715 × expression of *ATG101*) + (1.521474 × expression of *MAP1LC3C*) + (0.641122 × expression of *TSC1*) + (0.243022 × expression of *DAPK1*) + (-0.17015 × expression of *SERPINA1*). Based on the median risk score, all patients with colon cancer were divided into high- and low-risk groups. As displayed in Figures 3E, 5B, the high-risk score patients with colon cancer had a significantly increased mortality risk compared with the low-risk group. Figure 5 presents risk score of each patient (Figure 5A), survival status (Figure 5B) of each patient and the heatmap of 6 hub ARG expression (Figure 5C). Survival curves implemented by Kaplan-Meier methods demonstrated that in TCGA dataset, the high-risk score patients with colon cancer had a significantly shorter survival (*P* < 0.001) (Figure 5D).

To evaluate the prognostic accuracy of 6 ARG signature in TCGA colon cancer, the ROC analysis was implemented. As demonstrated in Figure 5E, the AUC of 6 ARG prognostic model for the prediction of 1-, 3-, and 5-year survival were 0.664, 0.697 and 0.711 respectively, indicating the potential robustness to predict the survival in colon cancer patients. PCA and t-SNE analyses demonstrated that the different risk score patients with colon cancer were discretely distributed in different directions (Figure 5F, 5G).

### The stratified analysis of different subgroups

**Figure 6:**
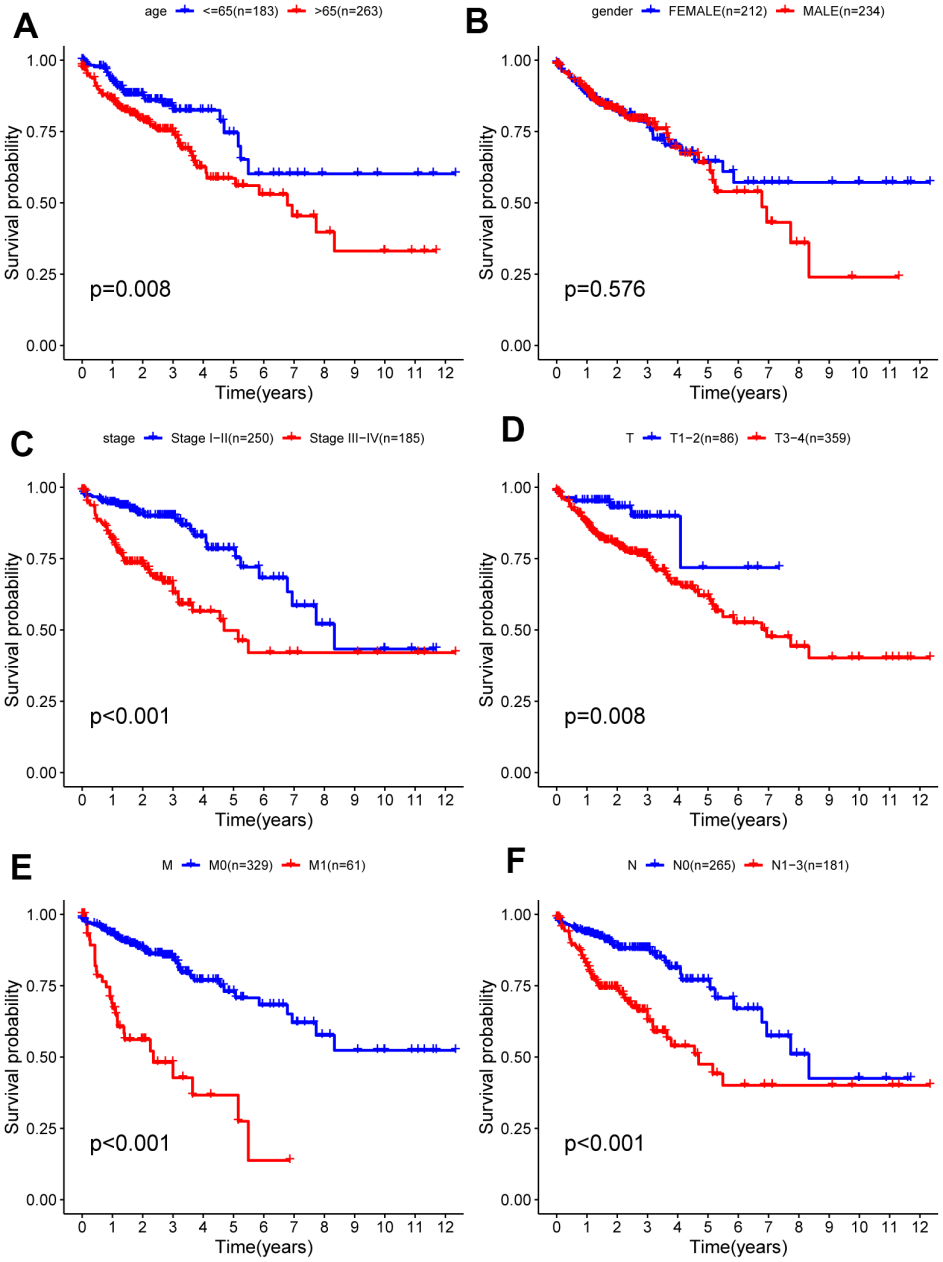
**Kaplan-Meier survival analysis of clinical features and survival rate.** Clinical features included (**A**) age, (**B**) gender, (**C**) stage (**D**) T, (**E**) M, (**F**) N.

**Figure 7:**
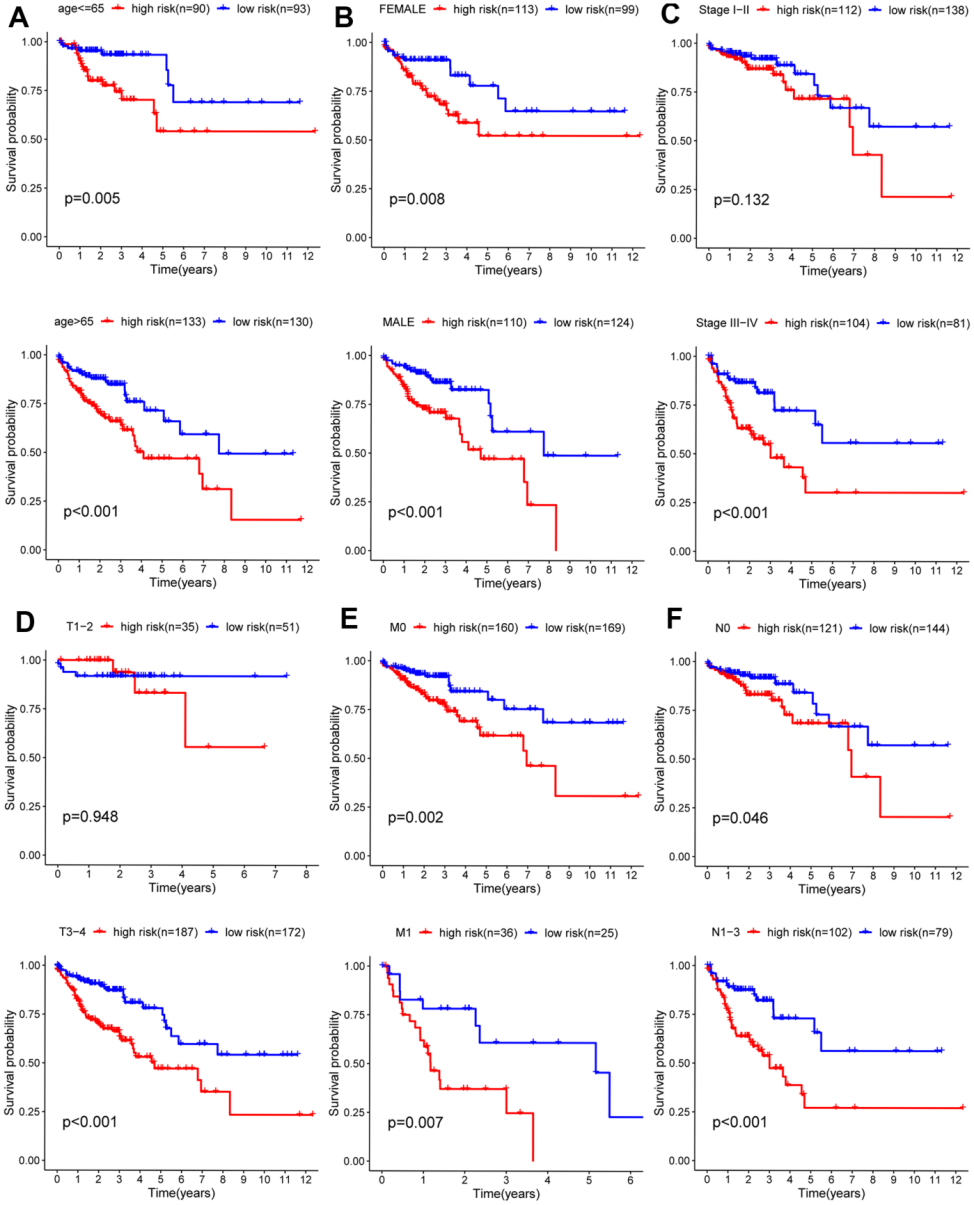
**Kaplan-Meier curves for prognostic value of risk-score signature for the patients divided by each clinical characteristic.** (**A**) Age, (**B**) gender, (**C**) stage (**D**) T, (**E**) M, (**F**) N.

Due to the unknown clinical data of some patients, relevant gene expression data were deleted. Analysis of the remaining 452 patients was carried out and correlations between clinical data and prognosis for survival were calculated, from which Kaplan-Meier curves were plotted. As shown in the Kaplan-Meier curves, age, T (primary tumor), M (metastasis), N (lymph nodes) categories, and tumor stage were statistically significant for prognosis (Figure 6). Subsequently, the stratified analysis of different subgroups was implemented. According to the Kaplan-Meier analysis, the model based on 6 hub ARGs had significantly distinct risk stratification ability in colon cancer. The results presented that the high-risk patients in colon cancer exhibited obviously worse prognosis. Whereas, in the Stage I−II (Figure 7C), T1-2 (Figure 7D) subgroups, this conclusion did not hold.

### Clinical correlation analysis

**Figure 8:**
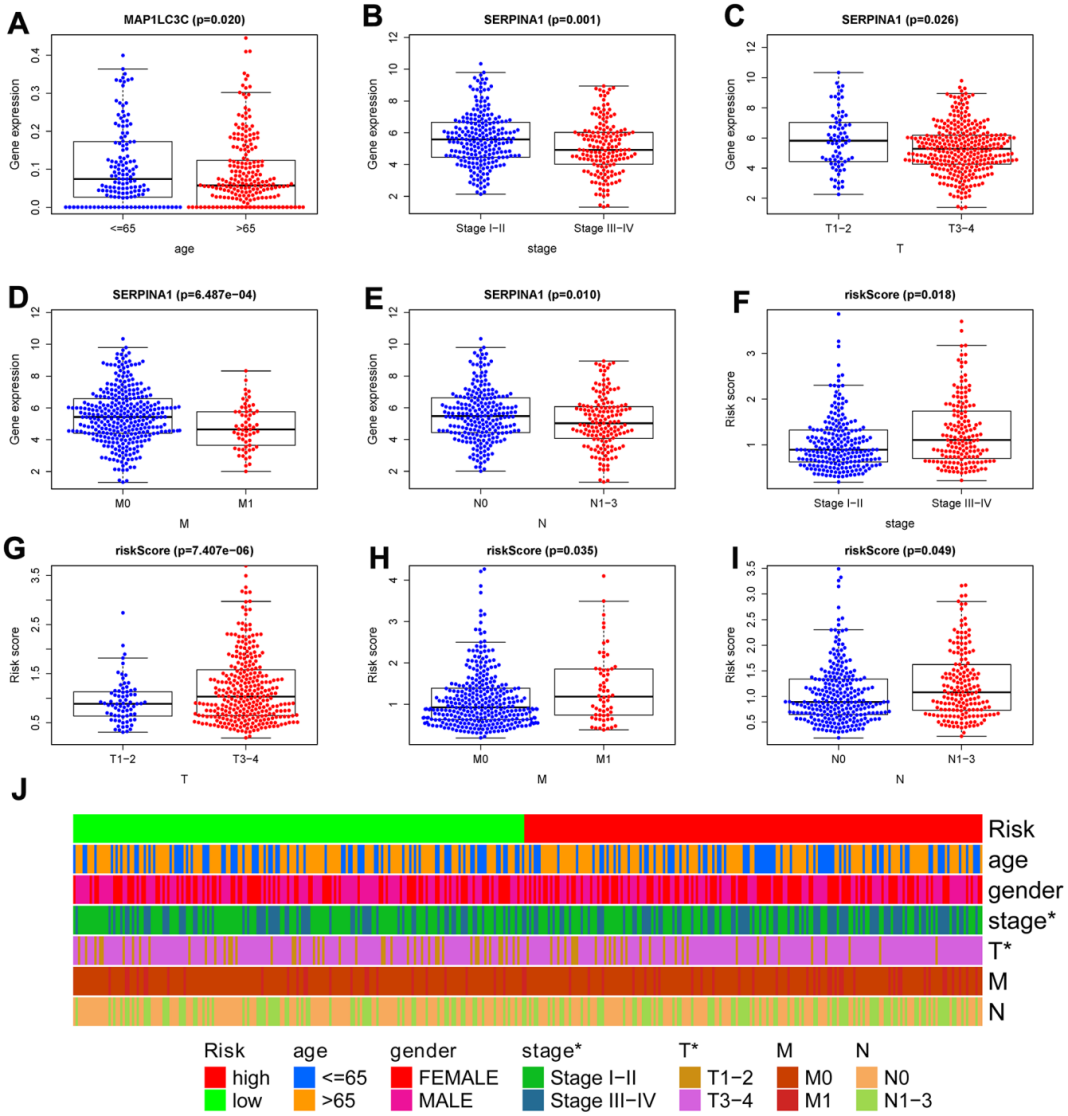
**Correlation between risk score signature and clinicopathological characteristics.** (**A**) RNA expression levels of MAP1LC3C in subgroups with different age; RNA expression levels of SERPINA1 in subgroups with distinct stage (**B**), T (**C**), M (**D**), N (**E**); risk-score in subgroups with different stage (**F**), T (**G**), M (**H**), N (**I**). (**J**) A strip chart showed the correlation between risk score signature and clinicopathological characteristics; *p < 0.05; **p < 0.01; ***p < 0.001.

To confirm the clinical value of the autophagy-related signature in colon cancer, we used the t-test to investigate the correlations among the 6 hub ARGs, autophagy-related signature, and clinical parameters. We found genes *SERPINA1* and risk score were significantly related to tumor stage, T, M, and N (Figure 8B–8I). Additionally, *MAP1LC3C* was significantly related to age (Figure 8A). Subsequently, we carried out a series of chi-square test to investigate the correlations between the autophagy-related signature and clinical features. The band diagram shows that tumor stage and T were significantly related to the autophagy-related signature (Figure 8J).

### Independent risk factors of OS and construction of a nomogram model

**Figure 9:**
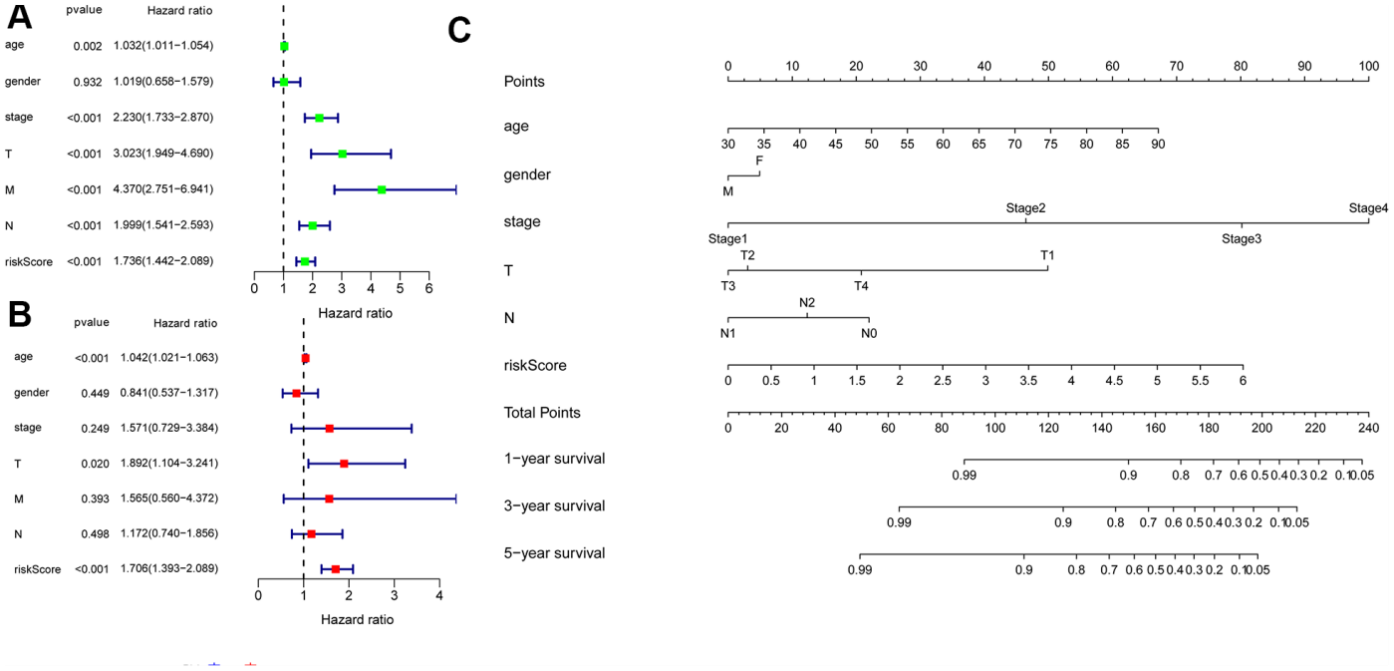
**Univariate and multivariate Cox regression analyses of OS and nomogram model.** (**A**) A forest plot of univariate. (**B**) Multivariate cox regression forest plot of independent risk factors. (**C**) An established nomogram model incorporated with the 6 gene signature and clinical factors for prediction of OS in patients with colon cancer in the TCGA dataset.

We combined clinical data with the risk score in colon cancer patients. The univariate Cox regression analysis revealed that age, tumor stage, T, M, N and risk score (all *P* < 0.05) were associated with survival (Figure 9A). Multivariate Cox regression revealed that age, T, and risk score (all *P* < 0.05) were independent risk factors for survival (Figure 9B). Subsequently, to develop a method for quantitatively assessing the survival time in colon cancer patients, we constructed a nomogram that incorporated risk scores based on 6 hub ARGs and clinical features (age, gender, T, N, and tumor stage) as shown in Figure 9C. In the nomogram, a dotted line is drawn with risk score and clinicopathological characteristics as parameters. The total score is obtained by adding the scores. The total score can be used to calculate the survival rate among colon cancer patients at 1, 3, and 5 years.

### Assessment of immune cell infiltration

**Figure 10:**
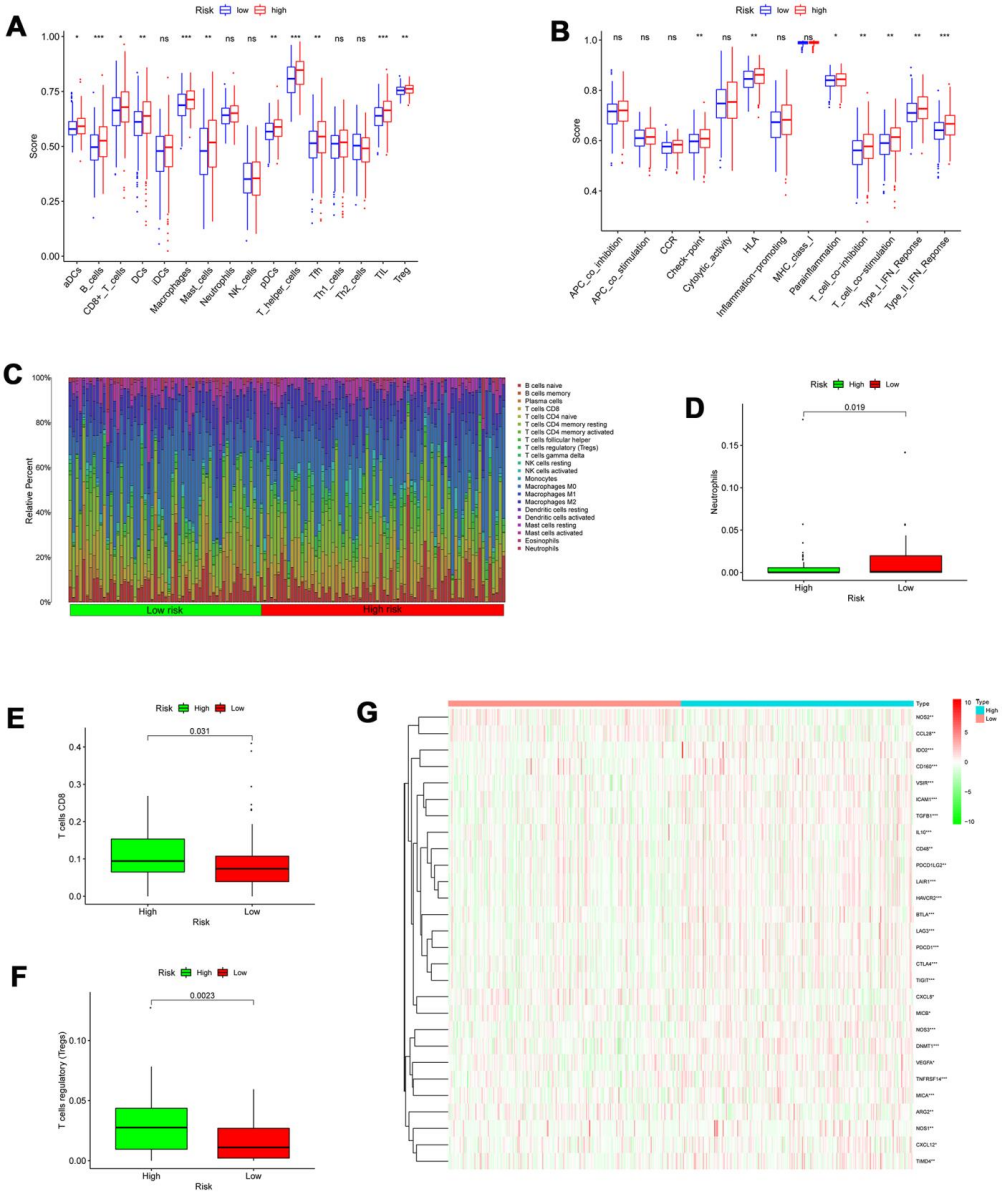
**Correlation between the risk signature and immune cell infiltration.** (**A**) Comparison of the infiltration of 16 immune cells between the different risk-groups. (**B**) Comparison of 13 immune-related functions between the different risk-groups. (**C**) The proportion of immune infiltration levels between the different risk-groups. (**D**–**F**) Box plots showing significantly different immune cells between the different risk-groups. (**G**) Heatmap of related negative genes involved in the regulation of the Cancer-Immunity Cycle between the different risk-groups; *p < 0.05; **p < 0.01; ***p < 0.001.

To evaluate the infiltration scores of immune cells and immune-related functions, we performed ssGSEA analysis to quantify the scores of immune cell infiltration and immunity-related functions. In the high-risk patients, antigen presentation cells (aDCs, DCs, and pDCs), B cells, CD8+T cells, TIL (tumor-infiltrating lymphocyte), macrophages, mast cells, T helper cells, Tfh cell, TIL (tumor-infiltrating lymphocyte), and T cells regulatory (Tregs) were significantly higher than in low-risk patients (Figure 10A). In the high-risk patients, the functions were at higher levels, including HLA (human leukocyte antigen), T cell co-inhibition, check-point, Type I/ II IFN response, and T cell co-stimulation (Figure 10B). Additionally, we utilized CIBERSORT algorithm to identify 22 types of immune infiltration cells for each sample. The significance threshold of the output result was set to *p* <0.05. The differences in 22 types of immune infiltration cells for each sample were evaluated in TCGA, representing features of personal differences (Figure 10C). As displayed in Figure 10D–10F, there were significant differences in neutrophils, T cells regulatory (Tregs), and T cells CD8 between the low- and high-risk score patients with colon cancer.

### Assessment of the immune-/chemotherapeutic response in the different risk-groups

A series of gradual steps, called the Cancer-Immunity Cycle, are needed to iteratively inspire and expand in order for the anti-cancer immune response to effectively kill tumor cells [17, 18]. The stimulus and inhibitory factors play a significant role in coordinately regulating every stage of the Cancer-Immunity Cycle. Here, we investigated the expression characteristic of genes negatively mediating the Cancer-Immunity Cycle between the high- and low-risk score patients with colon cancer. We downloaded these genes via Tracking Tumor Immunophenotype website (http://biocc.hrbmu.edu.cn/TIP/index.jsp). In the high- risk group, most of these genes were universally highly expressed as demonstrated in Figure 10G, suggesting that the high autophagy score patients are related to poor effect of immunotherapy.

**Figure 11:**
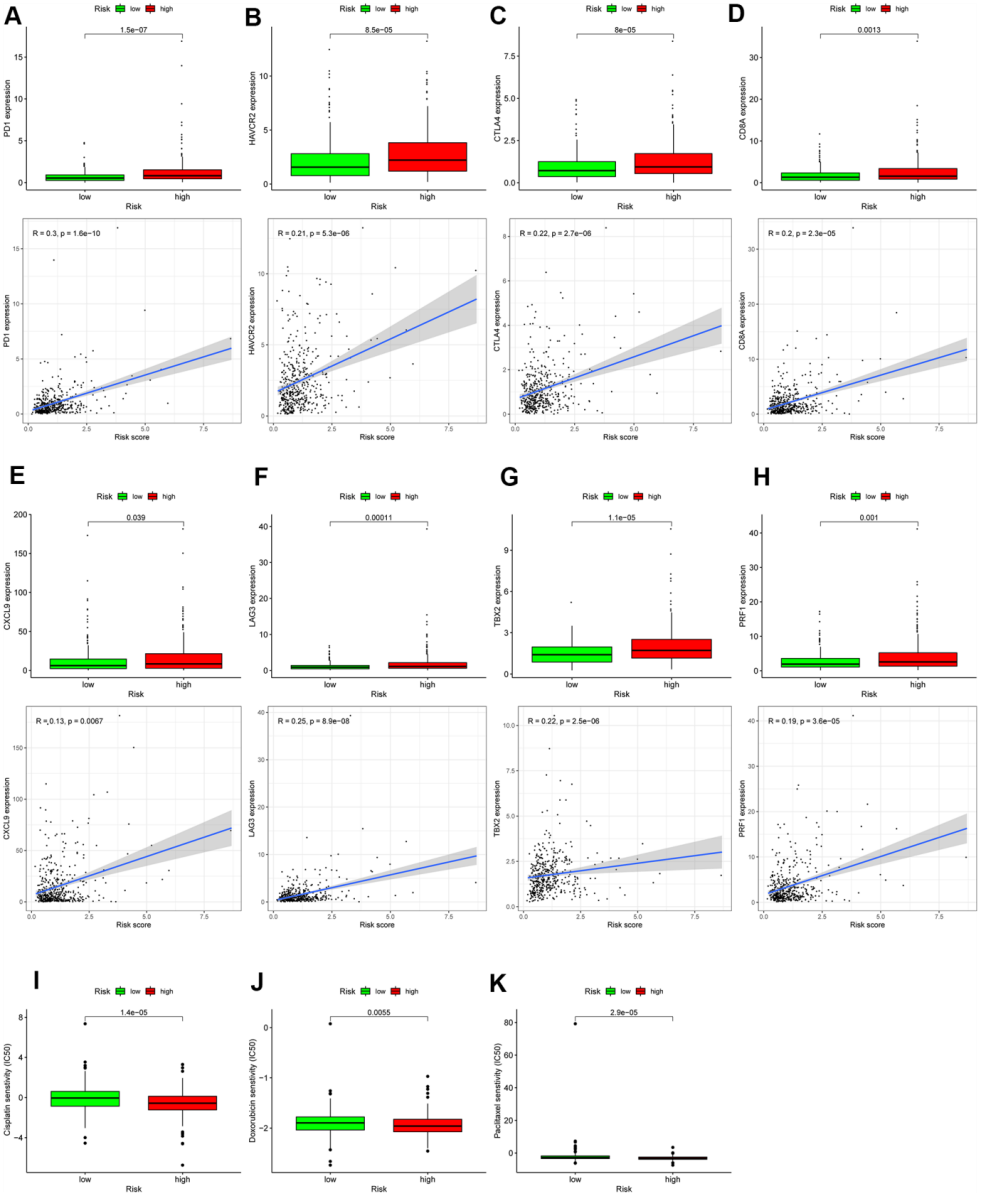
**Assessment of the immune-/chemotherapeutic response in the different risk-groups.** (**A**) *PDCD1*, (**B**) *HAVCR2*, (**C**) *CTLA4*, (**D**) *CD8A*, (**E**) *CXCL9*, (**F**) *LAG3,* (**G**) *TBX2,* and (**H**) *PRF1* expression between the different risk-groups and correlation between their expression and risk score. The risk score was negatively correlated with the IC_50_ of chemotherapeutics, including (**I**) cisplatin, (**J**) doxorubicin, and (**K**) paclitaxel; *p < 0.05; **p < 0.01; ***p < 0.001.

The previous evidence indicates that immune checkpoint therapy represented by specific ICIs, has achieved great immunotherapeutic efficacies in patients with colon cancer [19, 20]. Immune checkpoint therapy has been emerged as a new weapon against cancer. In view of the significance of it, further analysis on the relationship between autophagy-related risk score and expression level of genes related to ICIs is required. As displayed Figure 11A–11H, the expression of genes related to ICIs was positively correlated with the autophagy-related risk score and increased in the high-risk score patients with colon cancer (*P* < 0.05), such as *PDCD1, HAVCR2, CTLA4, CD8A, CXCL9, LAG3, TBX2,* and *PRF1.* The results above suggest that the high-risk score patients are more sensitive to immunotherapy. Chemotherapy is also recommended for advanced colon cancer treatment. The IC_50_ values of patients with colon cancer were calculated in accordance with the GDSC data. The autophagy-related score was negatively correlated with the IC_50_ values of chemotherapeutics in colon cancer, including doxorubicin (*P* = 0.0055), cisplatin (*P* < 0.001), and paclitaxel (*P* < 0.001) as demonstrated in Figure 11I–11K, indicating that autophagy-related risk score has potential predictive value for chemosensitivity in colon cancer.

### Validation of 6 ARGs expression

**Figure 12:**
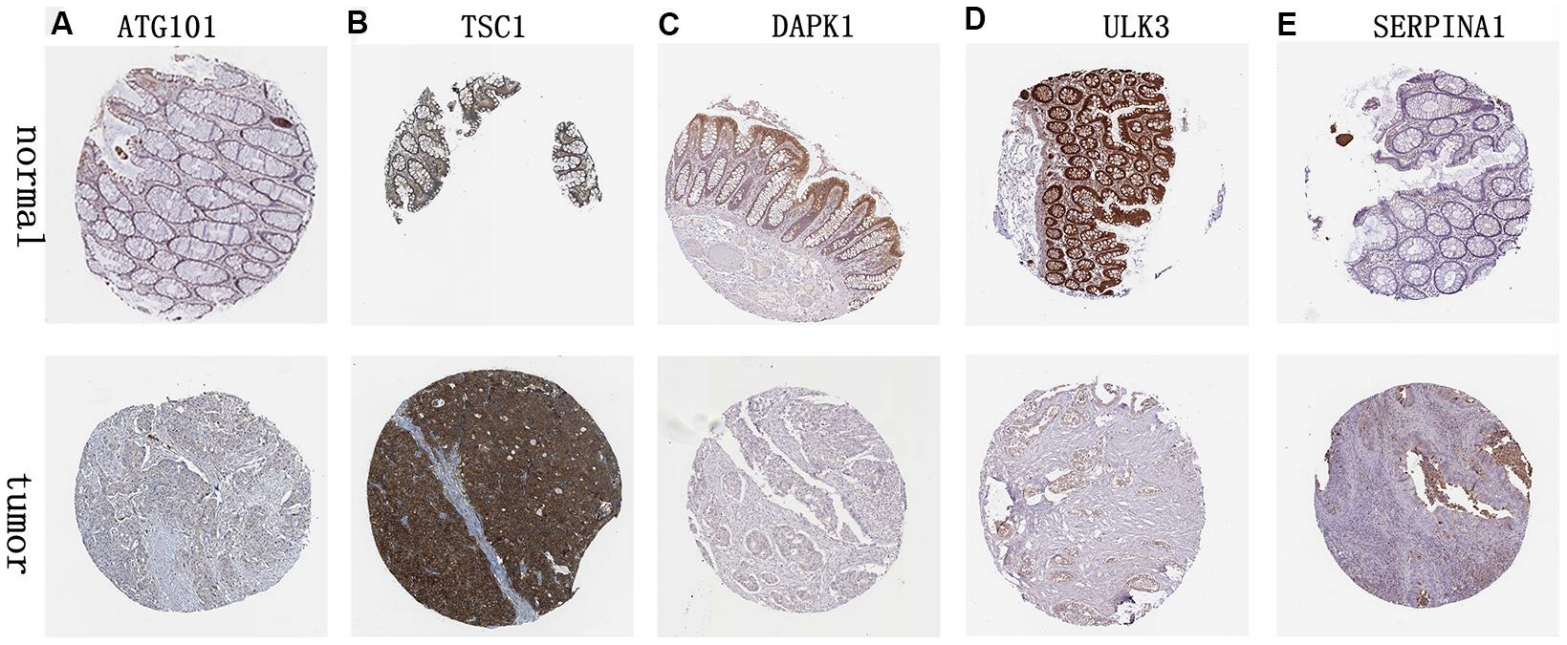
**Validation of hub ARG expression in colon cancer and normal tissue.** (**A**) *ATG101*, (**B**) *TSC1*, (**C**) *DAPK1*, (**D**) *ULK3*, and (**E**) *SERPINA1*.

We analyzed immunohistochemistry results of the Human Protein Atlas (HPA) database to investigate the expression levels of 6 hub ARGs in colon cancer. The results suggested that *ATG101* (Figure 12A) and *TSC1* (Figure 12B) levels in colon cancer tissues were significantly higher than normal tissues. Besides, the staining level of *DAPK1* (Figure 12C) and *ULK3* (Figure 12D) were reduced in colon cancer. However, there was little difference in *SERPINA1* staining levels between normal and tumor tissues (Figure 12E). The result of *MAP1LC3C* expression was not detectable.

## DISCUSSION

Colon cancer is characterized by an adverse survival rate and high recurrence rate [21]. It is of necessity to screen out sensitive and specific molecular biomarkers [22]. Although accumulating evidence indicates that autophagy acts as a crucial part in the malignant progression of colon cancer, ARGs have not been systematically analyzed to investigate their prognostic value in colon cancer. At the same time, genome sequencing technology is developing rapidly. Therefore, under the current circumstances, it is of importance to develop an autophagy-related model to predict the survival and prognosis in colon cancer patients.

This study first screened and identified 6 hub OS-related ARGs based on the TCGA dataset, and further established an autophagy-related signature. The present study demonstrated that the autophagy-related model including 6 ARGs could stratify the prognosis of colon cancer patients, and that high-risk score patients had poor overall survival. The autophagy risk scores also have similar results among colon cancer patients in different clinical features subgroups. As the stage and tumor grade increase, the autophagy risk score increases. These results indicate that the autophagy-related signature has a high and stable predictive value in the prognosis, and clinical features of colon cancer. Therefore, the new signature based on ARGs helps to identify high-risk patients, thereby helping to formulate efficient therapeutic plans for colon cancer patients, which is credible in clinical application.

Tumors can stimulate immune checkpoint targets to protect themselves from attack because immune checkpoints can promote tumor immunosuppressive effects [23]. Therefore, ICIs have achieved great immunotherapeutic efficacies by enhancing the immune system's killing effect on tumors [19, 20, 24]. In this study, the expression of genes related to ICIs was increased in the high-risk score patients with colon cancer, such as *PDCD1, HAVCR2, CTLA4, CD8A, CXCL9, LAG3, TBX2,* and *PRF1*. In addition, in the high autophagy-related risk patients, most of the genes negatively mediating the Cancer-Immunity Cycle were highly expressed, which also further promoted immunosuppression. The high autophagy risk group tends to form an immunosuppressive microenvironment by up-regulating immunosuppressive cytokines and immune checkpoints, and then becomes insensitive to immunotherapy. The autophagy-related score was negatively correlated with the IC_50_ values of chemotherapeutics in colon cancer, including cisplatin, doxorubicin, and paclitaxel, the result indicates that autophagy-related risk score has potential predictive value for chemosensitivity in colon cancer.

We screened out 6 hub ARGs significantly associated with OS, and several of the 6 hub ARGs have previously been demonstrated to be associated with autophagy and malignant tumors progression including colon cancer [25–28]. As another key node in autophagy, *ATG101* is involved in the formation of the complex (*ULK1–ATG13–FIP200–ATG101*) [29]. *ATG101* forms a heterodimer with *ATG13* via a single HORMA domain to contribute to stabilizing *ATG13* and *ULK1* in the complex [30]. *ATG101* has been demonstrated to be involved in the autophagy of human pulmonary arterial endothelial cells [25]. *TSC1-TSC2* (hamartin-tuberin) complex could negatively regulate the mTOR signaling pathway associated with autophagy [31]. TG or GG genotype of *TSC1* [27] have been demonstrated to be used as a potential therapeutic target to predict the worse overall survival or disease-free survival in colorectal cancer. Up-regulation of *ULK3* is involved in cancer-associated fibroblasts transformation and induces autophagy [28]. *DAPK1* (Death-associated protein kinase one) involves the phosphorylation of Beclin-1, which promotes Beclin1 dissociation from Bcl-X(L) and autophagy induction [32, 33]. The high expression of *DAPK1* in cholangiocarcinoma can reduce autophagy induced by cholangiocarcinoma cells and promote apoptosis of cholangiocarcinoma cells [34]. In diffuse large B-cell lymphoma, *DAPK1* is a promising prognostic and/or predictive marker of non-germinal central B-cell–like subtype, significantly reducing DFS and OS [35]. Jiang L. et al. reported that in gastric cancer, *SERPINA1* can regulate TGF-β signaling pathway to accelerate the growth and progression of tumor, indicating that *SERPINA1* may be a novel candidate therapeutic target [36]. The serpin α1-antitrypsin (AAT), encoded by the *SERPINA1* gene, may be a novel target for tumors to resist autophagic cell death [37, 38]. In colorectal cancer, expression of *SERPINA1* was positively correlated to survival, stage, N, furthermore *Snail* and *SERPINA1* have been demonstrated to promote colorectal cancer progression through fibronectin [26]. This is consistent with our findings that *SERPINA1* could serve as a biomarker to predict the overall survival in colon cancer. *MAP1LC3* acts as a significant part in the process of autophagosome generation [39], and is also involved in inducing vesicle expansion and interfering with the initial steps of membrane bending [40].

Notably, this study has some strengths. We have systematically analyzed the autophagy genes in the national database, which provides robust statistical support. Our study firstly established colon cancer autophagy-related prognostic scores, showing a high predictive value. The prediction of individual patients in the clinic has crucial guiding significance. Inevitably, several limitations are present. First of all, in view of the retrospective nature of the study, there is potential for inherent biases. Secondly, the autophagy-related signature for prediction, derived from the TCGA database, needs further validation in more cohorts to assure the effectiveness and robustness of the risk signature. Last but not least, some functional investigation is needed to further reveal the mechanism and prediction value of these 6 parameters in the future.

In summary, our current study systematically investigated the expression profile and clinical characteristics of ARGs in the TCGA database. We identified 6 prognostic-related ARGs (*ULK3, ATG101, MAP1LC3C, TSC1, DAPK1* and *SERPINA1*) to formulate a novel autophagy-related signature, which could stably predict the survival of patients and indicate the extent of immunosuppressive microenvironment in colon cancer. In addition, further prospective research on these genes may be beneficial to molecular targeted therapy of colon cancer and contribute to individualized efficient therapeutic strategies for colon cancer patients.

## Supplementary Material

Supplementary Figures
