# Supplementary material for: Development and validation of prognostic model based on the analysis of autophagy-related genes in colon cancer
Source: Aging (Albany NY). 2021 Jul 27;13(14):19028–47. doi: 10.18632/aging.203352 (PMC8351728; doi:10.18632/aging.203352)
Supplement: Supplementary Figures [file aging-13-203352-s001.pdf]

SUPPLEMENTARY FIGURES

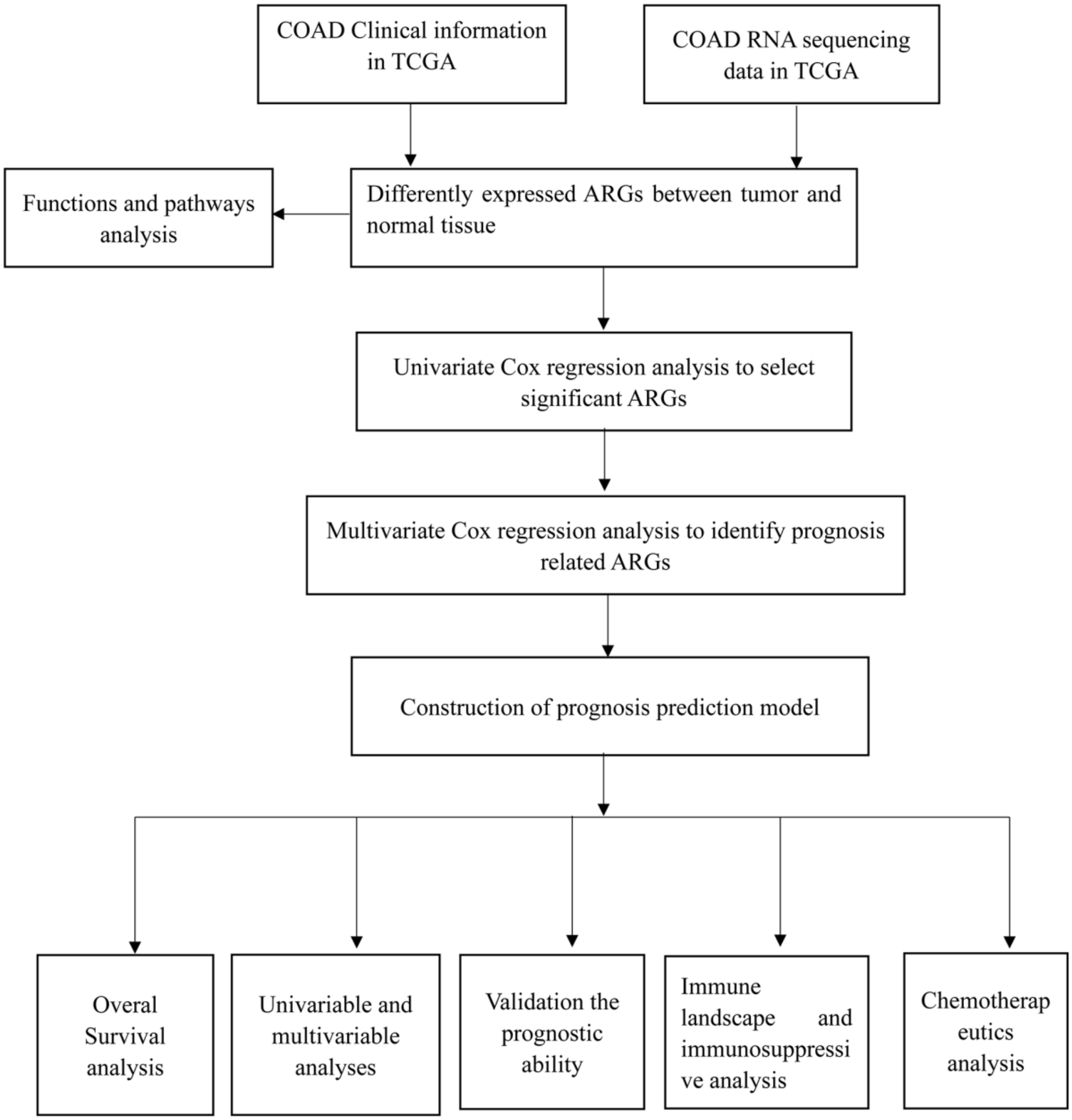

Supplementary Figure 1. Flowchart for identifying 12 ARGs signature associated with colon cancer survival.

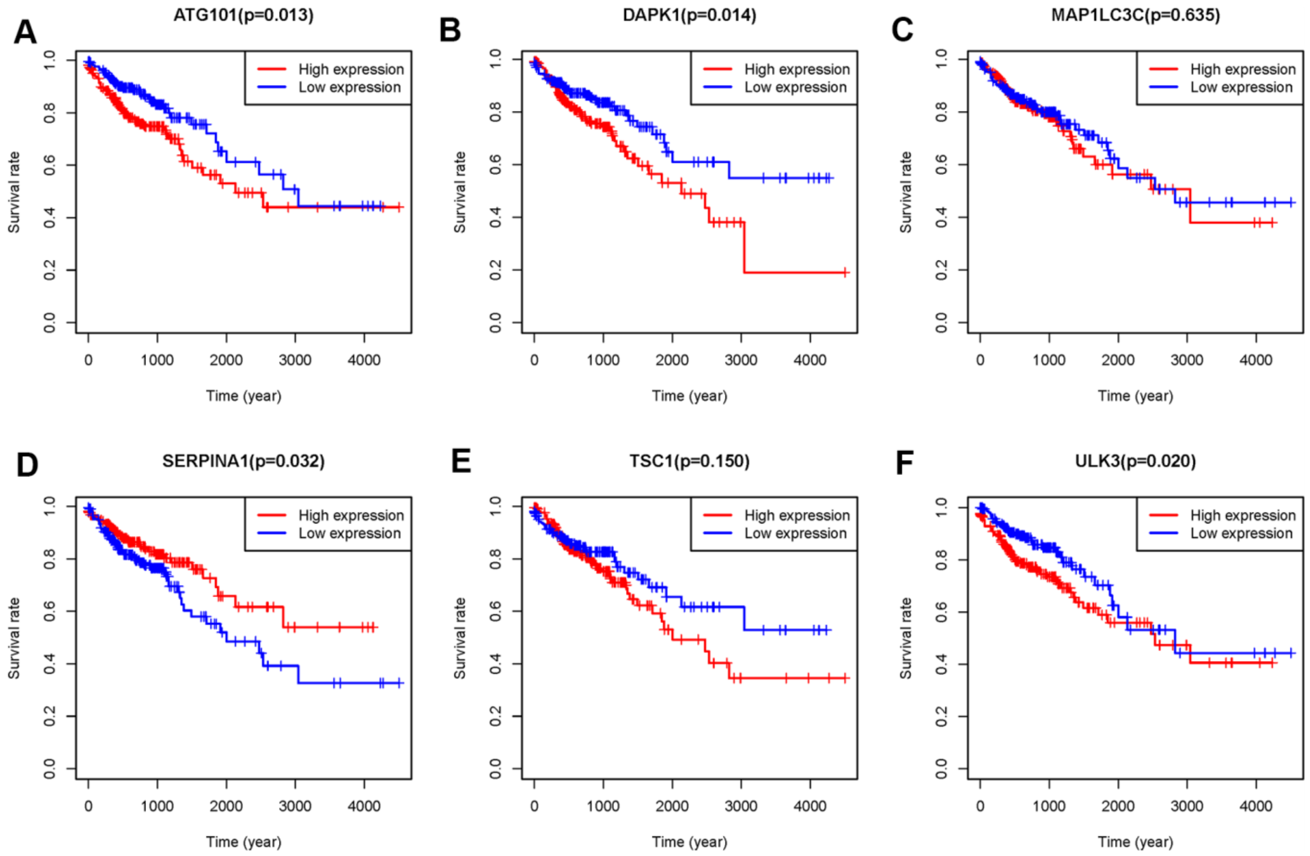

Supplementary Figure 2. Validation the prognosis value of 6 ARGs in colon cancer by Kaplan-Meier-plotter. (**A**) ATG101, (**B**) DAPK1, (**C**) MAP1LC3C, (**D**) SERPINA1, (**E**) TSC1, (**F**) ULK3.
